# The biomechanical signature of tumor invasion

**DOI:** 10.1016/j.gendis.2025.101771

**Published:** 2025-07-14

**Authors:** Chenhe Liu, Shijiang Wang, Xin Zhang, Yifan Han, Min Tan, Jiehou Fan, Jing Du, Yubo Fan, Xinbin Zhao

**Affiliations:** a Beijing Advanced Innovation Center for Biomedical Engineering, School of Biological Science and Medical Engineering, Beihang University, Beijing 100191, China; b Department of Radiation Oncology, Shandong Cancer Hospital and Institute, Shandong First Medical University, Shandong Academy of Medical Sciences, Jinan, Shandong 250117, China; c Beijing Advanced Innovation Center for Biomedical Engineering, School of Engineering Medicine, Beihang University, Beijing 100191, China; d Department of Breast Surgery, Second People's Hospital of Dezhou, Beijing 253000, China

**Keywords:** Actin remodeling, Mechanical forces, Mechanical memory, Microenvironment, Tumor invasion

## Abstract

Tumor cell invasion is the key driver of metastatic dissemination, resulting in the development and progression of metastatic tumors at secondary sites, and remains the major cause of cancer-related death. Recent studies suggest that, in addition to protease-mediated degradation and chemotaxis-stimulated migration, tumor invasion is significantly influenced by physical surroundings. How tumor cells decode information about their shape deformation under mechanical stress and adapt their dynamic behavior to escape the confined regions remains largely unknown. This review highlights recent findings that illustrate mechanical cues in confined tumor microenvironment contribute to tumor progression. We also systematically discuss the role of compression-induced deformation in cell membrane topology and cytoskeletal remodeling, as well as its biophysical mechanisms in regulating tumor invasion from a biomechanical perspective.

## Introduction

There were nearly 20 million new cases of cancer in 2022, alongside 9.7 million deaths from cancer from global cancer statistics based on updated estimates from the International Agency for Research on Cancer.^1^^,^^2^ Due to the impact of the COVID-19 epidemic over the past years, the closure of medical institutions has led to reduced access to medical opportunities and delays in diagnosis and treatment, which has contributed to a short-term decline in cancer incidence, however, the rate of late-stage cancer progression still rises, leading to an increase in mortality. Although significant progress has been made in tumor surgery, radiotherapy, chemotherapy, molecular targeted therapy, and immunotherapy, tumor invasion and metastasis have still not been prevented. A tumor is a mass of abnormal tissue formed when cells in a local tissue lose growth control at the genetic level, driven by various carcinogenic factors, resulting in abnormal clonal proliferation. The natural growth process of a typically malignant tumor includes the malignant transformation of a cell, clonal hyperplasia of transformed cells, local infiltration, and finally, distant metastasis. The main cause of death in cancer patients is tumor metastasis, and tumor invasion is the initial step in the development of tumor metastasis.^3^, ^4^, ^5^, ^6^ Acquisition of an aggressive phenotype is the most important characteristic of cancer and distinguishes malignant tumors from benign tumors.^7^, ^8^, ^9^

Most tumors that originate from epithelial cells are referred to as carcinomas. During the clinical pathological diagnosis process, researchers reported that cells with epithelial properties proliferate abnormally and further evolve into a tumor. Currently, the abnormally proliferated epithelial cells cannot yet infiltrate surrounding tissue, so it is a good window for treatment.^10^ Consequently, a more comprehensive and systematic understanding of invasive lesions at the early stage is conducive to early detection, diagnosis, and treatment of tumors, to improve the cure rate of various malignant tumors.

**Figure 1:**
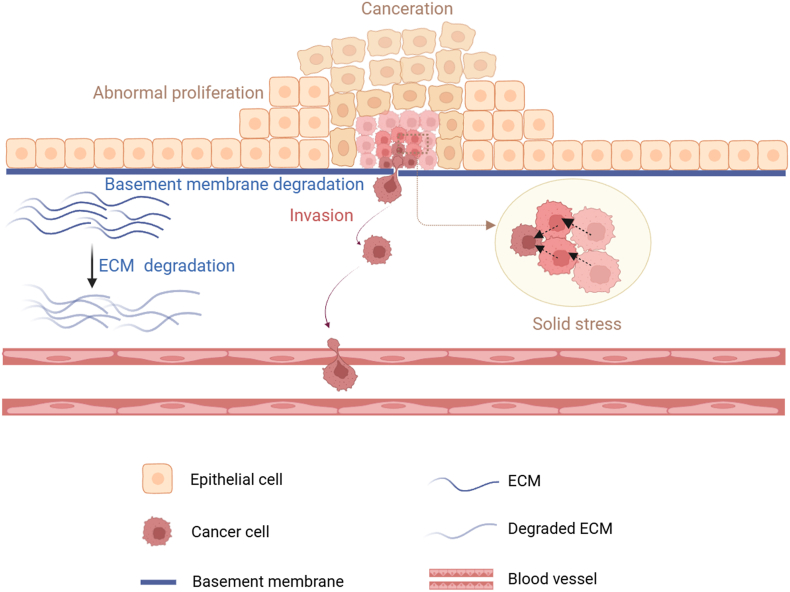
Tumor cell invasion process. As tumor cells abnormally proliferate and grow, they squeeze each other and undergo physical deformation because of mechanical forces such as solid stress. In the crowded microenvironment, tumor cells undergo epithelial-mesenchymal transition by secreting various cytokines and chemokines, at the same time, secreting metalloproteinases to degrade extracellular matrix (ECM) and promoting the invasion of tumor cells.

The degree of tumor infiltration is related to the invasiveness of tumor cells, which depends on the tumor cells and their microenvironment. The basement membrane of epithelial tissue mainly maintains the polarity and structural homeostasis of the epithelium, and is also an important barrier to prevent tumor cell metastasis. A sign of tumor invasion is the degradation of the basement membrane and extracellular matrix (ECM) (Fig. 1). In this process, tumor cells first form actin-rich invadopodia, release matrix metalloproteinases, destroy the basement membrane, dissolve the ECM, and finally complete the invasiveness of the tumor.^11^, ^12^, ^13^, ^14^ Therefore, clarifying the mechanisms of tumor invasion and infiltration has important guiding implications for tumor prevention and therapy.

**Figure 2:**
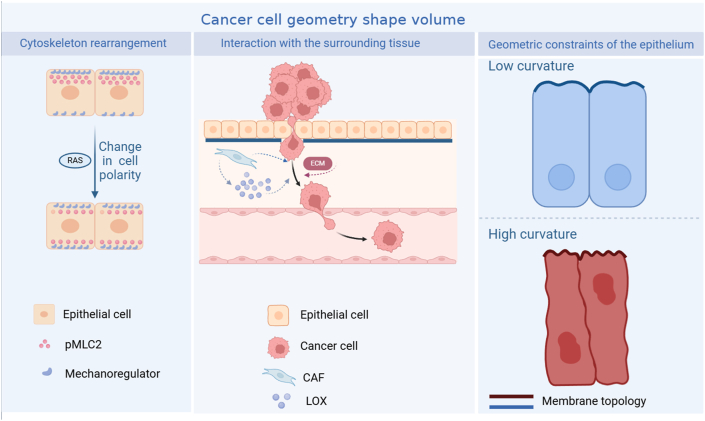
Architecture of tumor morphology. Proto-oncogenes-driven cytoskeletal rearrangements, geometric constraints of epithelial cells, and the tumor microenvironment jointly shape the morphological structure of tumor cells and their tissues.^16^^,^^139^ CAF, tumor-associated fibroblast; LOX, lysyl oxidase; pMLC2, phospho-myosin light chain 2.

In recent years, studies have shown that increased tumor volume, which is caused by tumor cell proliferation and ECM deposition, pushes and displaces existing viscoelastic structures inside and outside the tumor and generates solid stress in the tumor and the surrounding tissue.^15^ Tumor invasion is often accompanied by an increase in solid stress caused by the abnormal proliferation of tumor cells squeezing each other (Fig. 2). Meanwhile, the increase in solid stress induces changes in the topological structure of the tumor tissue.^16^ Cell surface topology profoundly influences biological behaviors such as cell adhesion, differentiation, and migration. Numerous scientific studies involving biomaterials have demonstrated that the change of nanoscale topology leads to the internal rearrangement of the cytoskeleton, thereby causing a series of cellular dynamics in living cells.^17^ At the same time, the deformation of the cell membrane mediated by the cytoskeleton is inevitably accompanied by changes in cell membrane curvature and tension.^18^ Cells can sense the microenvironment of cell membrane curvature and tension, allowing them to quickly switch between sustained and exploratory migration to avoid obstacles.^19^ Tumor tissue curvature and apical-basal mechanical tension imbalance induce epithelial tumor morphogenesis.^20^ Therefore, the topological morphology of cell surfaces profoundly affects tumor progression, including cell migration and invasion.

In conclusion, considerable progress has been made in understanding the molecular mechanisms and biological implications of tumors during the initiation and evolution stages of malignancy. In solid tumors, invasion and metastasis account for more than 90 % of the mortality rate. However, among the current anti-cancer treatments, there is a lack of effective anti-invasive and anti-metastasis drugs. The solid stress-induced cell extrusion because of the abnormal proliferation of the tumor *in situ* has long been confirmed.^21^ Previous studies demonstrated that changes in tumor tissue topology could affect the occurrence and development of tumors. However, in crowded tumor tissues, the exact biomechanical mechanisms of membrane deformation and cytoskeletal rearrangement in regulating tumor invasion remain unclear. Based on the above information, clarifying the biomechanical transduction is beneficial to a more systematic and comprehensive understanding of the complex regulatory network in the tumor microenvironment, and is important for further research on how the mechanical microenvironment in the tumor affects the process of tumor occurrence and development.

## Tumor invasion in the primary tumor

**Figure 3:**
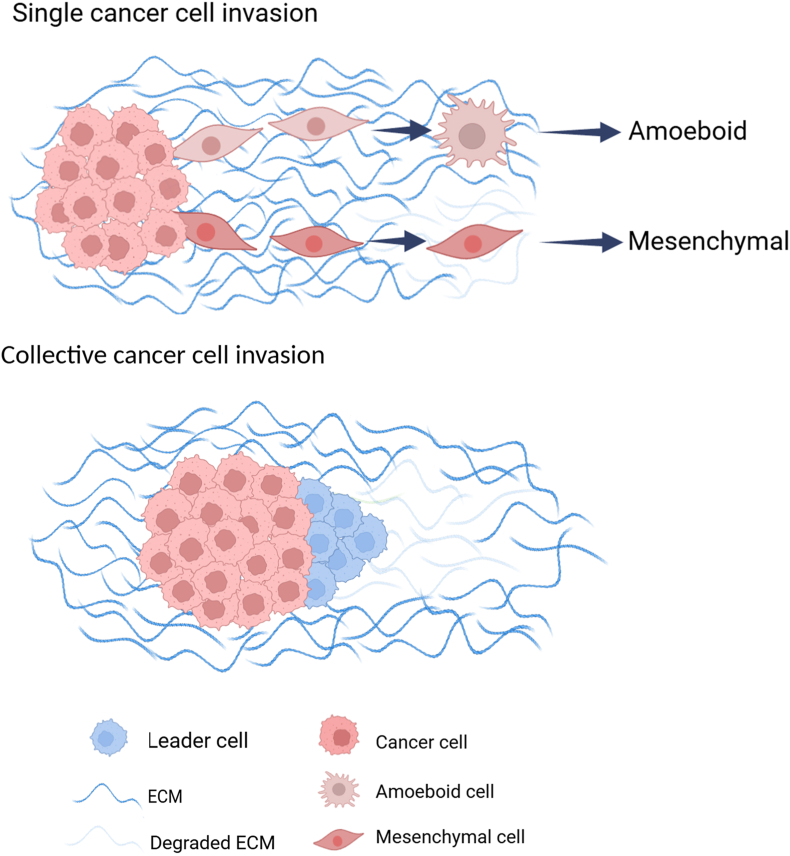
Modes and mechanisms of tumor cell invasion. For single cell invasion, the characteristics of amoebic invasion are circular and highly deformed cell morphology, with bleb-like protrusions and Rho-associated kinase-dependent contraction of myosin. The lack of ability in intracellular adhesion and extracellular matrix (ECM) degradation weakens the interaction between cells and the surrounding matrix. The characteristics of mesenchymal invasion are slender cell morphology, with cytoskeletal contractility, integrin-mediated matrix adhesion, and peripheral protein hydrolysis. For collective cell invasion, it maintains high intracellular adhesion and front-rear polarity, relying on actin dynamics, integrin-mediated cell adhesion to ECM components, and ECM reorganization mediated by extracellular protein hydrolysis.^140^

Tumor invasion is the key driver of metastatic dissemination, leading to the occurrence and development of metastatic tumors at secondary sites, which is still the main cause of cancer-related death.^22^^,^^23^ The activation of tumor invasion is one of the primary hallmarks of tumor transformation to malignancy, involving multiple biological processes, including changes in cell morphology, cell polarity, and cell body translocation.^24^ Tumor invasion is also one of the earliest steps in a series of phenotypic events that eventually lead to metastatic spread of the tumor, which includes malignant tumor cells breaking away from the site of origin, acquiring a plastic phenotype to actively move and invade the surrounding normal tissue by single cell invasion or collective cell invasion (Fig. 3). Although single cell invasion is the primary mode of entry into the blood vessels and lymphatic system, collective cell invasion is the main form of invasion and spread in most solid tumors.^25^^,^^26^

Epithelial-mesenchymal transition (EMT) is a cellular process during which epithelial cells acquire mesenchymal phenotypes and lose epithelial features, including apical-basal polarity, cell adhesion, and cell-cell junctions.^27^ The role of EMT in promoting tumor infiltration is instantaneous and reversible, which is the first step for tumor cells to spread outward and infiltrate into adjacent vascular systems.^28^, ^29^, ^30^ Recent studies have shown that the hybrid EMT state has stronger invasion and metastasis abilities than the complete mesenchymal state, and is more conducive to the generation of malignant phenotypes.^31^^,^^32^ Moreover, by identifying EMT transcription factors in various tumors, the hybrid EMT state plays a key role in collective cell invasion. In pancreatic ductal adenocarcinoma, ZEB1 is expressed in head and neck squamous cell carcinoma, and SNAIL is involved in behaviors such as collective cell migration.^33^ In breast cancer cells, the leader cells in a hybrid EMT state participate in the collective invasion because of the high expression of TWIST1 and ZEB.^34^ In addition, a recent study has shown that a hybrid EMT state is acquired through the induction of tumor-associated fibroblast-mediated paracrine secretion of ZEB1.^35^ These transcription factors (TWIST, SNAI, and ZEB) sense the changes of mechanical force, such as matrix stiffness.^36^ TWIST1 is located in the cytoplasm by binding to G3BP2 at low matrix stiffness; high matrix stiffness activates the Lyn kinase to phosphorylate TWIST1 to prevent its binding to G3BP2, thus inducing TWIST1 to enter into the nucleus to induce EMT.^37^ At high stiffness, MGAT5 activity enhances focal adhesion turnover and increases galectin 3, which increases the expression of ZEB1 and subsequently induces EMT.^38^ SNAI1 can serve as the molecular signaling downstream of ILK activation to induce EMT in high matrix stiffness.^39^

ECM is the main structural component of the tumor microenvironment, composed of interconnected macromolecular networks that exist in various tissues.^40^^,^^41^ ECM not only serves as a structural support but also plays a key role in tumor invasion.^42^, ^43^, ^44^ Due to the durotaxis of tumor cells on ECM, integrins and focal adhesions can activate intracellular signaling via the cytoskeleton to regulate the invasion and migration of tumor cells.^45^, ^46^, ^47^, ^48^ In addition, short and abundant filopodia are associated with a more aggressive tumor phenotype.^49^ Fascin, an actin bundling protein in filopodia, promotes cancer progression by increasing cell motility in metastatic tumors.^50^ Furthermore, specific small-molecule inhibitors of fascin can inhibit tumor cell migration and metastasis in mouse models.^51^ On the other hand, tumor invasion and metastasis first depend on the degradation of the basement membrane. To degrade the basement membrane and ECM, it needs the formation of actin-rich invasive pseudopods, and then releasing matrix metalloproteinases (MMPs) and other proteases.^43^^,^^52^, ^53^, ^54^ Furthermore, tumor cells can also secrete plasminogen activator and heparinase to degrade the ECM, promoting tumor invasion and migration.^40^ It has been shown that, in skin cancer, non-invasive basal cell carcinomas form “buds”, while invasive squamous cell carcinomas initiate as “folds”. Basal cell carcinoma accelerates the assembly to create a softer basement membrane. Squamous cell carcinoma reduces basement membrane assembly and imposes higher stiffness on it, inducing cell invasion.^55^

## Mechanical cues of tumor invasion

**Figure 4:**
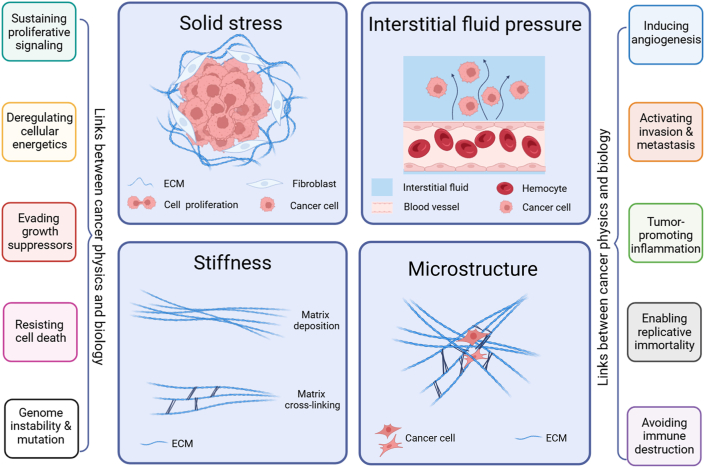
Biophysical traits of the tumor. The four major mechanical properties of tumors are solid stress (compression and tension), interstitial fluid pressure, matrix stiffness, and organizational microstructure. The physical and biological characteristics of the tumor interact synergistically.^15^

Mechanical properties of tumor microenvironment: Tumor is a heterogeneous, dynamic disease. In the tumor evolution, due to the rapid proliferation of tumor cells, uncontrolled growth of tumor volume, and changes in the composition and structure of the surrounding ECM, the mechanical microenvironment of the tumor undergoes dramatic changes, presenting different mechanical environments and characteristics in various regions. The current research proposes four distinct physical traits that arise from physical abnormalities in tumors (Fig. 4), including:

(i) Elevated solid stress (compression and tension). Solid stress is generated from the solid components of surrounding tissues being pushed and stretched by proliferating and migrating cells. Unlike liquid pressure that approaches zero in most normal tissues, solid stress is large enough to compress the blood and lymphatic vessels inside and around the tumor, act at the organ, tissue, and cellular levels, activate signaling pathways, and promote tumor invasion.
(ii) Elevated interstitial fluid pressure. Elevated interstitial fluid pressure is caused by plasma leakage from abnormally permeable tumor blood vessels and insufficient lymphatic drainage. Interstitial fluid leaks out of the tumor into the surrounding tissue, promoting invasion and metastasis through shear stress induced by blood flow.
(iii) Increased stiffness and altered material properties. The deposition and remodeling of ECM cause increased stiffness. The increase in stiffness activates signaling pathways that enhance tumor cell migration, invasion, and metastasis.
(iv) Altered tissue microstructure. When the normal tissue structure is disrupted by tumor growth and invasion, the microstructure is altered. The remodeling of ECM alters the interactions between tumor cells and their surrounding matrix, which affects signaling pathways related to invasion and metastasis.

The synergistic effect of the four physical characteristics above has a profound impact on many biological characteristics of the tumor, promoting tumor cell proliferation and invasion, immune system evasion, and resistance to treatment.^15^^,^^56^, ^57^, ^58^, ^59^, ^60^, ^61^, ^62^, ^63^, ^64^, ^65^, ^66^

### Compression features of tumor invasion

**Figure 5:**
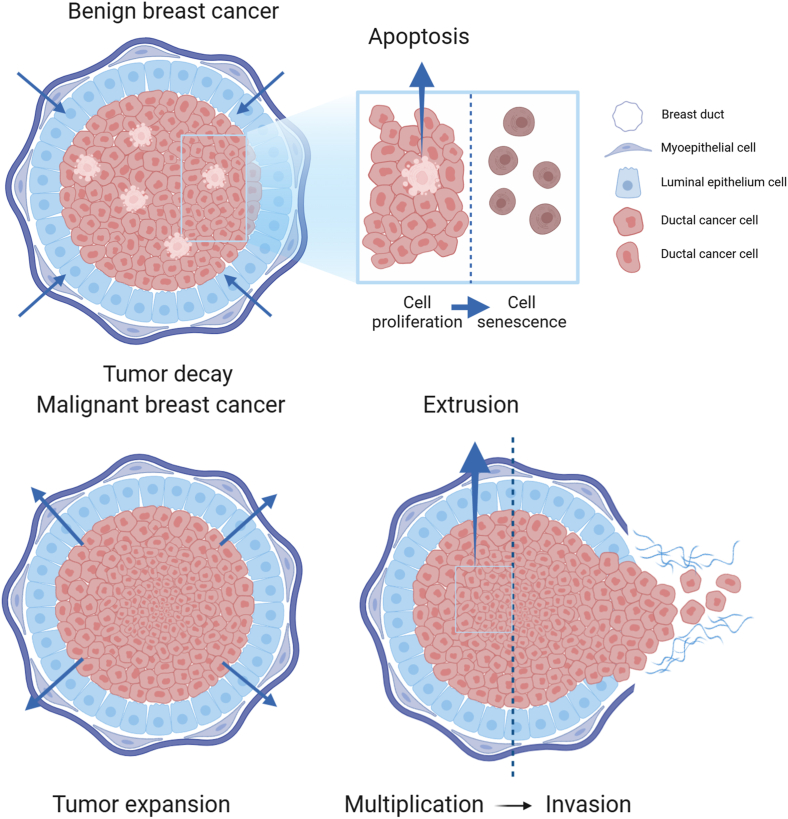
The occurrence and development of benign and malignant breast cancer. When the benign breast cancer cells proliferate to a certain number, tumor cells undergo apoptosis, and the tumor eventually goes into decline after the different components of the immune system become active. For malignant breast cells with abnormal proliferation and infinite expansion, tumor cells adapt their dynamic behavior to escape the crowded physical surroundings by invasion and metastasis.^74^^,^^141^^,^^142^

Since the 19th century, pathologists have known that changes in tumor cell shape in crowded tumor environments are key diagnostic features of advanced tumors, but the impact of these changes on cancer progression remains unclear. On the one hand, the study found that during the occurrence and development of malignant tumors, the mechanical characteristics of the frontier edge in tumor invasion are more significant and complex. For example, breast tumors exhibit high heterogeneity in matrix stiffness and collagen structure. The central cancer cells are abundant and quite soft, the collagen fibers at the forefront of invasion are arranged, and the matrix stiffness is high. The stiffness of the matrix in the invasive front area of liver cancer tissue is significantly higher than that of the central tumor tissue. Tumor cells infiltrating the marginal zone may include non-proliferative tumor cells that may undergo EMT, which has strong motility and invasiveness.^46^^,^^67^ On the other hand, many studies recently showed that abnormal tumor proliferation led to a crowded microenvironment within tumor tissue, which affected the life activities of tumors and promoted the invasion and metastasis of tumor cells.^68^ In 2015, Matthieu Piel and Verena Ruprecht found that physical confinement and low adhesion induced rapid amoebic migration of mesenchymal cells, suggesting that tumor cells could spontaneously escape primary sites and invade surrounding tissues without any specific genetic alteration.^69^ Cortical contraction force can drive embryonic progenitor cells to acquire fast and persistent migratory motility.^70^ In 2020, Matthieu Piel and Verena Ruprecht further pointed out that the nucleus could sense the compression on cells and respond to these mechanical challenges in their tissue microenvironment.^71^ Under overcrowded conditions, actin contraction of cells can be triggered to assist tumor cells in escaping the confined environment.^72^ Meanwhile, Hosseini et al found that tumor cells failed to divide because of confined space and underwent invasion to escape the crowded environment.^73^ In addition, Matthieu Piel revealed that overcrowding due to physical compression could cause DNA damage and subsequently induce the migration of tumor cells and accelerate tumor infiltration.^74^ In summary, large numbers of studies recently show that excessive compression caused by abnormal proliferation of tumor cells in very narrow spaces can trigger a series of signaling pathways and biological regulation, causing tumor cells to squeeze out and escape crowded environments, leading to invasion and metastasis (Fig. 5).

### Basal extrusion and tumor invasion

**Figure 6:**
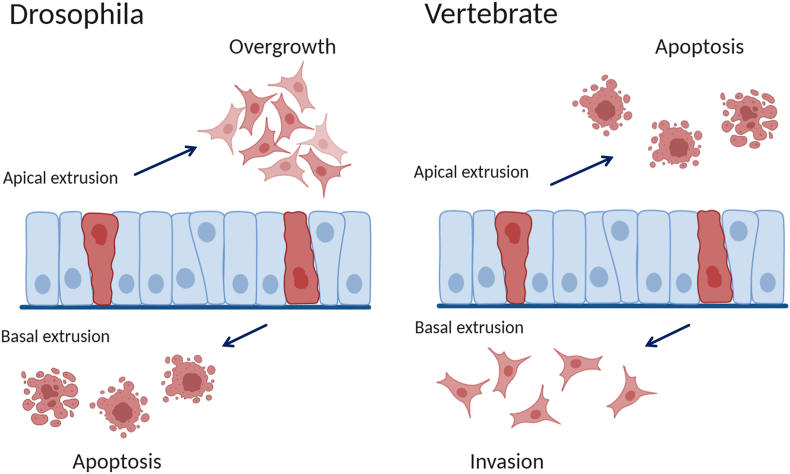
Extrusion direction determines the fate of tumor cells. Epithelia remove live or dead cells in response to cell stimulation caused by crowding or apoptosis, respectively. For *Drosophila*, a small part of anti-anoikis cells can overgrow within their lumen by apical extrusion, despite that most of them eventually die because of anoikis. Meanwhile, basal extrusion can lead to cell apoptosis. For vertebrates, basal extrusion can cause invasion of extruded cells, compared with apical extrusion-induced cell apoptosis.^143^^,^^144^

A crucial primary step for cancer metastasis is invasion, but we know very little about the mechanisms of invasion. As metastasis is the major cause of treatment failure in cancer patients and of cancer-related deaths, understanding the mechanisms that drive invasion will be crucial for studying targeted drugs for malignant tumors. Considering that it is difficult to directly track the invasion of tumor cells from the primary sites, there is no intuitive data to capture and elaborate on this process. To understand how tumor cells invade, it is helpful to study the migratory behavior of normal epithelial cells. Epithelial cells form a selective protective barrier for all the tissues that they enclose. Polarized epithelial cells consist of an apical surface facing the lumen (external environment) and a basal surface facing the basement membrane. Epithelial cells are the first line of defense against pathogens and toxins; therefore, these cells that constitute epithelial tissue are exposed to potential damage. As a result, many epithelial cells constantly turn over due to cell division and death. When epithelial cells become too crowded because of proliferation, to maintain epithelial homeostasis, epithelia can remove surplus cells from overcrowded regions by extruding live or dying cells and inhibit the occurrence of tumors. However, recent studies found that carcinogenic mutations could prevent epithelial cells from extruding from the top, but instead induce cells to extrude from the base beneath the epithelial cells (Fig. 6). In this way, basal extrusion enables cells with higher survival and proliferation potential and allows extruded cells to invade the underlying matrix.^62^^,^^75^

## Cell membrane topology in tumor invasion

The topological morphology of cell surfaces profoundly affects biological behaviors such as cell adhesion, differentiation, and migration. A series of scientific studies focused on biomaterials demonstrated that nanoscale topological changes can influence the dynamic assembly of surface structures and the rearrangements of the actin cytoskeleton in living cells.^17^^,^^76^^,^^77^

### Topological morphology of tumor cell surface

**Figure 7:**
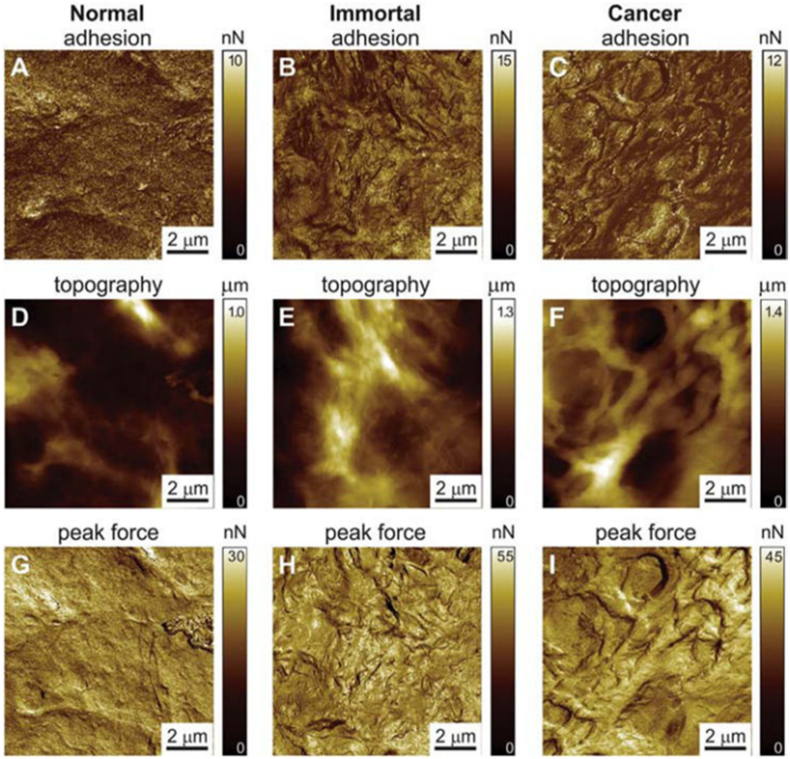
Surface topological morphology of human cervical epithelial cell carcinoma at different stages.^145^ Representative examples of 10 × 10 μm^2^ adhesion maps and height images of normal, pre-malignant, and malignant cells by atomic force microscopy. Normal cells look smoother, and malignant cells have more wrinkled surfaces in the adhesion. The brighter means the higher value of either adhesion or height.

Before invasion, both the surface structure and intrinsic volume of tumor cells undergo physical changes due to the influence of mechanical factors, such as cellular deformation caused by solid stress compression. Given the complex and irregular shape of tumors, researchers used fractal geometry to simulate skin cancer and calculated the fractal dimension of skin cancer tissue as a diagnostic tool for the aggressiveness of skin cancer cells.^78^ Because of the complex anatomical structure and cellular characteristics of normal lung tissue, the morphological changes caused by tumor growth will inevitably have a significant impact on the fractal dimension of lung tissue. Therefore, the fractal dimension of tumor tissue can be used for clinical imaging research related to tumor growth, diagnosis, and treatment.^79^ Researchers have performed fractal analysis on the surface of fixed and dried cell membranes using atomic force microscopy imaging to obtain a physical adhesion map between atomic force microscopy probes and cell surfaces (Fig. 7). It is proved that fractal dimension can be used to separate normal cells, cervical cancer precursor cells, and cervical cancer cells.^80^ Furthermore, a previous study showed that the fractal geometry on the surface of cervical cancer cells was effective at a specific stage of transformation from precancerous cells to cancer, and before or after this stage, cells did not show a strong correlation with fractals.^81^ There was also emerging evidence from studies demonstrating that the Minkowski dimension, by calculating the boundary between tumors and normal tissues, could be used to determine the invasiveness of cancer cells. As a quantitative measurement method for cancer diagnosis, the value of Minkowski dimension increased along with the augmentation of cell invasiveness.^82^

### Cell curvature

The changes in cell membrane topology are mediated by the actin cytoskeleton, usually followed by alteration of membrane curvature and tension.^18^ During tissue morphogenesis, a rapidly growing epithelial flake shows a smooth and continuous curvature on both the apical and basal sides. The tangential force on the apical surface maintains smooth curvatures by preventing irregular fluctuations during tissue morphogenesis. However, in rapidly growing epithelial cells, the irregular pushing forces caused by cell transition division produce substantial mechanical disturbances, disrupting the balance between global and local forces and causing dramatic changes in cell curvature.^83^ Recent studies demonstrated that membrane curvature was no longer seen as a passive result of cellular activity, but rather as an active means of creating membrane domains and controlling membrane transport. Curvature can dynamically regulate cell movement through changes in lipid composition, oligomerization of membrane scaffold proteins, and conformational changes in transmembrane regions of proteins.^84^, ^85^, ^86^ Cells can sense the surrounding microenvironment by membrane curvature and tension, allowing them to quickly switch between persistent and exploratory migration to circumvent obstacles.^19^

### Curvature and tension of tumor cells

**Figure 8:**
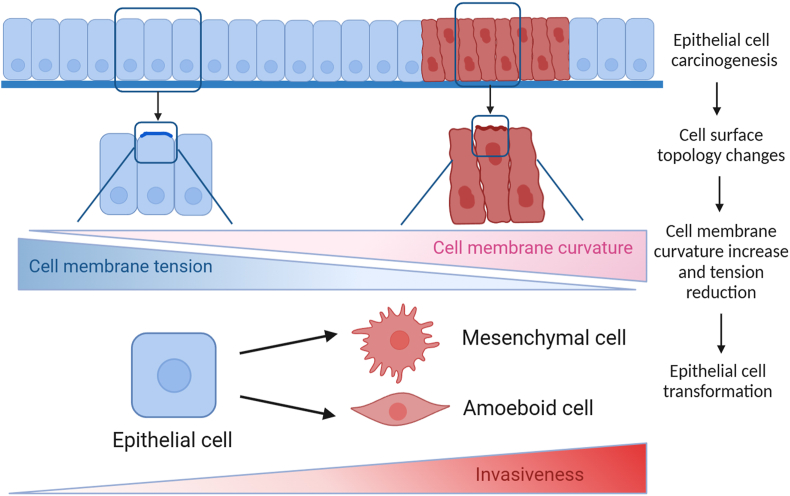
The effect of cell membrane topology on tumor invasion. Epithelial cell carcinogenesis and abnormal proliferation induce cell crowding by physical compression, causing tumor cell deformation and changes in surface topology. The increased membrane curvature and the decreased membrane tension between the apical and basal sides drive cancerous epithelial cells to transform into invading amoebic or mesenchymal cells.^146^

The curvature changes of tumor tissue or the imbalance of mechanical tension between the apical and basal membrane can induce the morphogenesis of epithelial tumors.^20^ In the pancreas, the heterogeneous geometric shape of the pancreatic duct affects the early morphogenesis of pancreatic ductal adenocarcinoma and its precursor lesions. The carcinogenic transformation of pancreatic ducts can lead to endophytic lesions that grow into the lumen of ducts with a diameter of 17 μm. The higher curvature of narrower ducts can also drive early tumors to grow exophytically into the surrounding parenchyma.^16^ Using artificial geometries and lung alveolospheres derived from human pluripotent stem cells, researchers found that tumor cells could perceive curvature at the multicellular scale and respond to regulate their collective migration. As the curvature of the monolayer increases, the collectivity of the cell decreases, and the multicellular flow field is more dynamic, which becomes less collective for cell migration.^87^ Epithelial cells can maintain higher plasma membrane tension than metastatic cancer cells (Fig. 8). The high membrane tension effectively inhibits the migration and invasion of cancer cells by resisting the Bin/Amphiphysin/Rvs (BAR) family proteins that sense membrane curvature.^88^ In addition, the high expression of EMT-inducing transcription factors can reduce plasma membrane tension to promote tumor cell migration and invasion.^88^

### Actin remodeling and tumor invasion

Cell invasion is influenced by various extracellular stimuli, involved in many signaling pathways. Actin polymerization and myosin contraction are necessary for driving cell invasion and migration.^89^ Furthermore, actin structure is highly dynamic and remains constantly remodeling in living cells. The dynamics are based on a highly controlled equilibrium of local assembly and disassembly of actin filaments, mainly regulated by the Rho and Rac GTPase subfamilies.^90^, ^91^, ^92^ Microfilaments in cells lengthen and shorten by the addition and loss of subunits at their ends, and branched microfilaments, as daughter filaments, can be separated from the mother filament by debranching factors. Branched microfilaments are composed of the Arp2/3 complex, triggered by WASP family proteins, and bind to the side of a mother filament. Actin is also involved in the assembly of multimolecular focal complexes: lamellipodia, a dense and branched network involved in cell protrusions; filopodia, a finger-like structure at the leading edge of the motile cell, composed of aligned microfilaments, sensing the extracellular environment, and affecting the direction of cell movement; and stress fibers, dynamic structures composed of a large number of antiparallel actin filaments.^93^ In tumor cells, microfilaments are attached to the plasma membrane by plasma membrane–microfilament linkers, thereby controlling the shape of the cell membrane and inhibiting the excessive expansion of the protrusions at the membrane. Additionally, cell invasion can be driven by localized cell membrane protrusions via the directional polymerization of actin in invadopodia.^94^

## Mechanical mechanisms of actin remodeling-mediated tumor invasion

### Actin remodeling at the apical side of the cell

The cortical actin layer at the top of the cell, located underneath the apical membrane, is a mesh-like structure of membrane-proximal F-actin and actin-binding proteins.^95^^,^^96^ The plasma membrane binds to the microfilament of the cell cortex via the linker proteins.^94^ The changes in the organization, thickness, and contractility of the cellular cortex, composed of microfilaments, can affect cell shape and play important roles in cell signal transduction and cell migration.^97^ Membrane protrusions and cell migration are regulated by the density of F-actin close to the plasma membrane. Furthermore, nanoscale topology affects membrane curvature by the membrane curvature-sensitive protein FBP17, mediating the participation of the Arp2/3 complex in the assembly of branched actin.

### BAR family proteins

**Figure 9:**
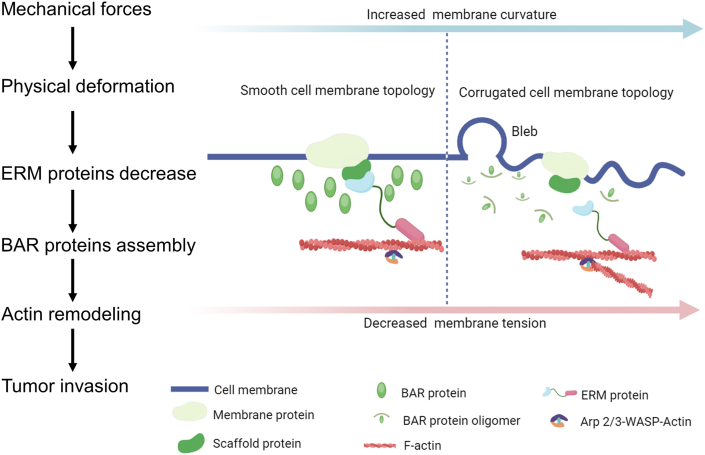
The proposed model describing how cell membrane topology and cortical actin act as the mechanical sensor of tumor invasion. As membrane curvature increases and tension decreases, the membrane topology changes from smooth to corrugated. At the same time, the ERM proteins connecting the plasma membrane and cortical actin dissociate from the membrane, and the BAR protein polymerizes from monomer/dimer to oligomers. Eventually, cortical actin filaments are converted into branched actin by the Arp2/3-WASP complex.^88^ Actin remodeling leads to tumor cell invasion by decreasing ezrin/radixin/moesin (ERM) proteins and assembling Bin/Amphiphysin/Rvs (BAR) proteins.

BAR proteins are crucial for membrane organization, fission, dynamics, and tubule generation within the cells. The BAR domain possesses a characteristic crescent-like shape, enabling it to bind to and induce curvature in cellular membranes. BAR domain proteins can detect membrane deformations caused by external forces (*e.g.*, solid stress, stretching) and participate in numerous cellular processes by sculpting the membrane, including endocytosis, membrane trafficking, filopodia, and tubule formation.^98^ BAR proteins recruit effectors like small GTPases Cdc42 to regulate cytoskeletal remodeling and activate PI3K/Akt signaling pathways.^99^ FBP17, a BAR domain-containing protein regulating membrane curvature, acts as a sensor of membrane tension involved in actin-based directed migration.^100^ BAR proteins MTSS1L and Toca are implicated in lamellipodia formation and elongated invasive behavior through the activation of Arp2/3complex-dependent actin nucleation and the depletion of ezrin/radixin/moesin (ERM) proteins, respectively^101^^,^^102^ (Fig. 9).

### ERM proteins

ERM proteins are involved in tethering the membrane to the cellular actin cortex as well as regulating cortical organization and mechanics.^103^ In their active conformation, the N-terminal FERM domain binds to the cytoplasmic tails of transmembrane proteins, and the C-terminal ERM association domain region binds to actin filaments.^104^ Ezrin unfolds under shear stress, exposing binding sites for adhesion molecules (CD44) and actin, enabling force transmission from the ECM to the cytoskeleton. Ezrin integrates Rho-GTPase signaling and functions as both an upstream and downstream effector of Rho GTPases to regulate cytoskeletal organization, influencing cell stiffness, polarity, and invasion.^105^ Phosphorylation of ERM proteins stabilizes their active state, amplifying mechanical signals into biochemical outputs.^106^^,^^107^ Ezrin functions as a scaffold protein that recruits proteins involved in signal transduction and determines the survival of epithelial cells. The mechanism by which ERM proteins are involved in invasion and metastasis is their induction of EMT and interaction with CD44.^108^ A recent study shows that the plasma membrane-actin linker protein ERM can effectively stabilize the membrane tension to inhibit tumor invasion and metastasis, by counteracting the BAR protein activity caused by membrane curvature and blocking Arp2/3 complex-dependent cortex actin polymerization^88^ (Fig. 9).

### Actin remodeling at the basal side of the cell

**Figure 10:**
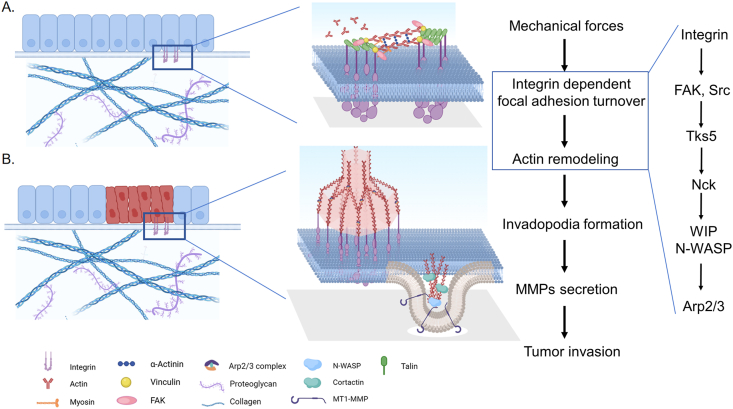
Molecular mechanisms of integrin-mediated actin remodeling in tumor invasion. The components of the actin complex in focal adhesions and invadopodia are similar, but their assembly, structure, and functions are completely different. Actin stress fibers are anchored to the plasma membrane at the integrin-dependent focal adhesions parallel to the extracellular matrix (ECM) (A), in contrast, the actin core in invadopodia is oriented perpendicular to the ECM and cell membrane (B). Invadopodia are built on a Tks5/Nck/WIP/N-WASP-dependent branched actin network, and Rho GTPase and Cdc42 drive pseudopodia-membrane protrusion, which is mediated by bundled actin filaments that form the actin core as the initial step in invadopodia assembly.^147^

Polymerization of actin cytoskeleton, changes of membrane curvature, switch of cell-matrix adhesion, and contraction of myosin may play important roles in invadopodia formation (Fig. 10). Except for matrix metalloproteinases degrading ECM in invadopodia, cortactin is required for coordinating the dynamics of membrane-forming invadopodia. As one of the cytoskeletal proteins for invasive cell assembly in cancer cells, cortactin regulates the remodeling of the actin cytoskeleton by activating the Arp2/3 complex.^109^^,^^110^ Instead of binding to focal adhesions directly, cortactin aggregates at the edge of lamellipodia and forms an actin cytoskeletal network to promote cell migration and invasion.^111^ Many studies have shown that cortactin overexpression induces tumor invasiveness by promoting the formation of invadopodia.^112^^,^^113^ Moreover, cortactin was proven as a substrate for Src, which is often highly expressed in cancer. Src can phosphorylate cortactin to enhance actin assembly and increase the binding force of cortactin to Nck/WIP protein, thereby facilitating the formation of new invadopodia.^114^^,^^115^ Further, the phosphorylation of cortactin interacts with WIP, which makes it stable instead of triggering its localization to invadopodia. Supervillin, microfilament, and myosin-binding proteins can also reorganize the actin cytoskeleton and enhance invasion by acting as intermediaries for cortactin 116.

Integrins are a structurally and functionally diverse family of 24 heterodimeric type I transmembrane glycoproteins, multi-protein complexes that link the intracellular actin cytoskeleton to the ECM. The ability of ECM signals to orchestrate the formation and function of invadopodia is based on mechano-chemical integrin outside-in signaling and requires integrin cross-talk. Multiple adhesion and contractility molecules localize to and around invadopodia, implying that the ECM rigidity response occurs directly at invadopodia.^117^ For invadopodia, the initiation step begins with the formation of the actin core. After that, integrins and associated adhesome components are recruited^118^ and form an adhesive ring around the actin core. Continued polymerization of the actin core is believed to push the invadopodia membrane outwards and drive its penetration into the surrounding ECM.^119^ With the number of proteins present at focal adhesions, numerous studies reveal that integrin signaling is involved in the processes of mechanical forces-mediated tumor invasion. β1 integrins have been found to regulate invadopodia by forming signaling complexes with Src, EGFR, and/or FAK.^120^ In addition, β1 integrin interacts with Arg, a tyrosine kinase, leading to invadopodia maturation and matrix degradation by activation of MT1-MMP, which aids in invasion through integrin-mediated Src and EGFR signaling.^121^

## 3D models of tumor invasion

Tumor invasion methods have been developed and are routinely used to evaluate the unidirectional invasion, such as transwell invasion assay. However, this is limited because of the challenges of 3D imaging. To address the limitation, advances in material fabrication techniques (*e.g.*, microfluidic devices, 3D bioprinting, and high-throughput robotic systems) are supporting the development of sophisticated 3D models of tumor invasion.^122^

### Spheroid invasion models

The *in vitro* tumor models are usually used to monitor the real-time invasiveness of cancer cells. Spheroids are formed using cell aggregation techniques, including the hanging drop method, cell pelleting, spinner flasks, and the liquid overlay method.^123^ Advanced spheroid invasion models apply functional materials, 3D bioprinting technology, and high-throughput robotic systems. A new tumor invasion model was developed for a high-throughput system using a custom-designed fabrication platform, which used an adapted hanging drop method to culture cells in 3D, directly allocating each spheroid into a 96-well plate after spheroid formation.^124^ A 3D bioprinting technique was developed to precisely locate spheroids in alginate hydrogels using a matrix ink and a sacrificial bio-ink. Tumor cells can be clustered in the empty space inside the matrix bio-ink and form spheroids after dissolving the sacrificial bio-ink.^125^

### Vascularization models

3D microfluidic platforms allow the characterization of individual cellular responses, providing intercellular communication through real-time visualization and the ability to study specific biological cues.^126^ These models have been developed to investigate metastasis-related phenomena, such as EMT, tumor cell migration and invasion, intravasation, and extravasation. The enhanced invasion of MDA-MB-231 cancer cells into the endothelial vascular network was observed using a microfluidic device designed with three channels: the tumor channel filled with MDA-MB0231 cells, the stromal region filled with collagen, and the vascular region composed of endothelial cells.^127^ Cui et al developed a breast cancer cell bone metastasis model using a 3D bioprinting technology. Human fetal osteoblasts and MDA-MB-231 cells were seeded onto the bone and tumor matrix, which were printed with a photo-cross-linkable GelMA ink. Endothelial cells were injected into the vessel-like channel, which was printed with GelMA ink between the bone and tumor matrix.^128^

### Mechanical memory and tumor invasion

Previous studies on mechanical memory have primarily focused on mesenchymal stem cells and epithelial cells. These cells were observed to retain their original phenotypic characteristics after mechanical stimulation on stiff substrates, even when transferred to softer environments.^58^ However, recent evidence of mechanical memory in tumor cells has begun to emerge. Epithelial cells, including non-tumorigenic MCF10A and oncogenic MCF-7 cell lines, retain the behavior acquired in the primary tumor even after their dissemination. When cultured on polyacrylamide gels mimicking stiff (50 kPa) or soft (0.5 kPa) matrix conditions and subsequently transferred to soft gels, cells initially exposed to the 50 kPa substrate maintained faster collective migration compared with those preconditioned on the softer matrix. Mechanical memory was retained because those cells primed on stiff substrates maintained YAP nuclear translocation even after transitioning to soft gels.^129^ In addition, mechanical memory is also found in breast cancer cells. When cultured on 8 kPa polyacrylamide gels for 7 days and then transferred to 0.5 kPa soft gels for 24 h, these cells preserved their highly invasive capacity. Research has demonstrated that matrix stiffness-driven cytoskeletal remodeling and ERK protein phosphorylation activate the transcription factor RUNX2, driving its nuclear translocation to promote tumor invasion.^130^

YAP and RUNX2, involved in mechanical memory, usually occur within minutes to hours of exposure to high matrix stiffness. For a longer timescale (days to weeks), mechanical forces can lead to epigenetic changes that include histone modification through DNA methylation, histone acetyltransferases, histone deacetylases, and non-coding RNAs. Previous studies showed that mechanical cues altered chromatin accessibility^131^ and transcriptional ability^132^ in normal epithelial cells. Although persistent epigenetic changes that potentially regulate mechanical memory have not yet been identified in tumor cells but have been found in mesenchymal stem cells,^133^^,^^134^ a recent study has shown that increased matrix stiffness can increase lamina-associated chromatin and there are more accessible chromatin sites binding to Sp1 transcription factor in cells embedded in 2 kPa 3D hydrogels than those in 0.1 kPa hydrogels,^135^ suggesting that phenotypic adaptations of tumor cell maybe retained via mechanical memory encoded by persistent epigenetic changes.

## Conclusions

Within the tumor microenvironment, tumor cells are exposed to various mechanical stimuli, including solid stress from the expanding tumor mass, interstitial fluid pressure, and shear stress. Mechanical forces in the tumor microenvironment can impinge on many steps of the metastatic cascade. In this review, we have conceptualized the invasion process in which tumor cell changes include dramatic tumor cell shape, mechanics, and motility in the tumor microenvironment. To escape a crowded microenvironment, tumor cells must deform to squeeze through confined spaces. The detailed molecular mechanism of nucleus deformation caused by compression begins to emerge, considering the large and relatively rigid characteristics of the cell nucleus. However, many unanswered questions remain surrounding the role of cellular perception and transduction from mechanical cues during tumor progression. A systematic and comprehensive analysis of the physical properties of cell membranes, combined with an improved understanding of mechanosensitive membrane proteins and signaling pathways, should also be taken seriously.

In particular, our current understanding of various mechanical signaling in tumor invasion is mostly based on 2D cell culture. The *in vitro* systems of cell invasion driven by mechanics fail to simulate and replicate the physiological microenvironment in humans, as well as the lack of cell-phenotypic measurements. Thus, developing models to study these mechanosensitive pathways in an *ex vivo* 3D platform can help us deeply investigate how exactly these physical parameters affect chemical signaling. With the advancement of micro/nanotechnology, we can better understand the physical interactions between nano-membrane topology changes and mechanical forces and the molecular mechanisms regulating cell invasion responses to mechanical stimuli. In summary, mechanics play critical roles during tumor progression, and new insights into mechanotransduction pathways in cancer will have significant implications for our fundamental understanding of tumor invasion.

The biomechanical signature of tumor cells has been implicated in the development and progression of cancers such as skin, breast, and lung cancer.^136^, ^137^, ^138^ Physical changes in the shape of tumor cells, including bending and protrusion, promote the migration and invasion.^88^ This review provides clues of cell-scale mechanics for potential clinical strategies targeting the biophysics of mechanosensitive proteins to prevent carcinoma invasion. In particular, given the essential roles of cell membrane topology and actin remodeling in tumor invasion, it is valuable to further investigate if small molecules or gene-targeting entities could be used to enhance the function and rescue the surface topology of tumor cells. These therapeutic interventions may suppress the progression of tumors toward a more malignant stage by normalizing the biomechanical signature of tumor cells subjected to mechanical stimulus.

## CRediT authorship contribution statement

**Chenhe Liu:** Writing – original draft. **Shijiang Wang:** Writing – review & editing. **Xin Zhang:** Writing – original draft. **Yifan Han:** Data curation. **Min Tan:** Software. **Jiehou Fan:** Visualization, Validation. **Jing Du:** Conceptualization. **Yubo Fan:** Project administration, Funding acquisition. **Xinbin Zhao:** Supervision, Conceptualization.

## Funding

This study was supported by the 10.13039/501100004826Beijing Natural Science Foundation (China) (No. L248062) and the 10.13039/501100012226Fundamental Research Funds for the Central Universities of China (No. KG16301601).

## Conflict of interests

The authors declared no conflict of interests.
